# Graph-Based Auditory Brainstem Response Threshold Estimation from Incomplete Intensity Series

**DOI:** 10.3390/bioengineering13101128

**Published:** 2026-09-27

**Authors:** Yuan Tao, Pengyu Ren, Peng Han

**Affiliations:** 1 Department of Otorhinolaryngology-Head and Neck Surgery, The First Affiliated Hospital of Xi’an Jiaotong University, Xi’an 710061, China; taop19772000@163.com; 2 Department of Otorhinolaryngology, Peking University Shenzhen Hospital, Shenzhen 518000, China; 3 Department of Neurosurgery, The Second Affiliated Hospital of Xi’an Jiaotong University, Xi’an 710004, China; reaping2006@gmail.com

**Keywords:** auditory brainstem response, hearing threshold estimation, stimulus intensity response graph, graph neural network, waveform morphology encoding

## Abstract

Auditory brainstem response (ABR) threshold determination requires clinicians to identify the transition between waveforms with and without a detectable response across stimulus intensities. Automation remains challenging because responses near the threshold are weak, stimulus series are often incomplete, and the interpretation of one waveform depends on the remaining series from the same ear. To address these challenges, we represent each ear as a masked stimulus intensity response graph and propose ABR-GraphDoseNet for automated ABR threshold estimation. The model encodes waveform morphology at each stimulus intensity, exchanges information across an ordered intensity graph, and models candidate threshold transitions with a decoder for adjacent nodes. A global threshold head further integrates evidence across the complete ear series. We evaluated 2064 ears from 1045 subjects using ten-fold cross-validation grouped by subject. ABR-GraphDoseNet achieved the best overall performance, with a mean absolute error of 0.90±0.27 dB, an exact accuracy of 93.80±1.41%, and an accuracy within 10 dB of 98.64±1.01%. Ablation experiments identified the global threshold head and morphology tokenizer as the largest contributors, while adaptive edges and the boundary decoder provided smaller complementary gains. Overall, ABR-GraphDoseNet provides an accurate, structured, and auditable framework for modeling incomplete ABR stimulus series. External validation across devices, acquisition protocols, and institutions remains necessary.

## 1. Introduction

Auditory brainstem response (ABR) is a short-latency scalp potential generated by synchronized activity along the auditory nerve and brainstem pathways after acoustic stimulation [1,2]. Because recording proceeds without an active behavioral response, ABR is central to objective hearing assessment in infants and in patients for whom behavioral thresholds are unavailable or unreliable [3]. Current early hearing detection guidance places physiologic assessment within the pathway from screening to diagnosis and intervention [4]. Click and tone burst ABR thresholds can predict clinically relevant regions of the behavioral audiogram, although the correspondence varies with stimulus frequency and degree of hearing loss [5]. In practice, the reader examines waveforms from one ear across several stimulus intensities and identifies the lowest level at which a repeatable response remains detectable [6].

Near-threshold ABR responses are weak relative to noise, and acquisition settings affect their morphology [7,8,9]. Threshold estimation, therefore, requires joint interpretation across stimulus intensities, as also reflected in probabilistic approaches [10]. Clinical series are often incomplete because preceding responses guide subsequent measurements. Unmeasured levels must be distinguished from measured weak responses, while observation patterns may reflect clinical workflow as well as physiology [11,12,13].

Automated ABR analysis has progressed through several methodological stages. Early systems translated expert latency and threshold rules into computer procedures [14], and descriptor vector methods subsequently framed response detection as a statistical classification task [15]. Support vector approaches coupled designed representations with explicit descriptor selection [16], whereas correlation and reproducibility criteria provided objective alternatives to visual inspection [17]. Later signal processing methods improved threshold decisions through subaverage comparison and response consistency [18], including real-time detection based on cross-correlation [19]. Machine learning classifiers then broadened the representation space used for response detection [20]. Subsequent studies compared these classifiers directly with established statistical detectors [21]. More recent supervised and self-supervised models further reduced dependence on manually specified descriptors [22].

Recent work moves from isolated response detection toward joint modeling of waveform morphology and the complete stimulus series. A systematic review found that machine learning studies of human auditory evoked responses vary in inputs, targets, and validation procedures [23]. Resampled cross-correlation across subaverages has enabled efficient automated thresholding while retaining an interpretable measure of response consistency [24]. Deep learning toolboxes can automate several ABR analysis steps, but their threshold decisions still depend on how the model represents information across levels [25]. Sequence models explicitly combine temporal waveform representations with stimulus intensity and align series patterns across levels [26]. Nevertheless, an incomplete series poses three unresolved representation problems: preserving physical intensity order, separating unmeasured levels from measured weak responses, and exposing the transition that supports the final threshold.

The threshold decision contains both local and global evidence. Locally, the decision defines a boundary between neighboring intensity levels where a reproducible response ceases to be detectable. Globally, the plausibility of that boundary depends on the morphology and progression across all observed levels. Graph neural networks provide a natural mechanism for propagating evidence over a predefined relational structure [27], and attention can learn which connected observations should contribute most strongly to each representation [28]. Attention-based sequence modeling also demonstrates the value of direct interaction among ordered observations [29]. However, existing ABR approaches rarely combine an explicit intensity graph, a local boundary decision, a global ear level decision, and a visible observation mask in one model.

To address these limitations, we represent each ear as a masked stimulus intensity response graph and propose ABR-GraphDoseNetfor automated ABR threshold estimation. In the model name, the term Dose refers to ordered acoustic stimulus intensity levels rather than medication dosage. Each nominal stimulus intensity corresponds to a fixed graph node, observed waveforms populate the corresponding nodes, and a binary observation mask identifies unmeasured levels. This representation preserves intensity order while explicitly separating measurement missingness from weak waveform content.

The main contributions of this study are as follows:

- We introduce a masked stimulus intensity response graph for each ear that preserves intensity order and distinguishes unmeasured levels from measured weak responses.
- We propose ABR-GraphDoseNet, which integrates waveform morphology, stimulus intensity information, relationships between adjacent stimulus levels, and adaptive graph interactions.
- We develop a local and global decision framework that jointly models candidate threshold boundaries and evidence distributed across the ear series.
- We evaluate the proposed method with ten-fold cross-validation grouped by subject, competitive baselines, component ablations, and error analyses by threshold.

## 2. Materials and Methods

### 2.1. Study Design

This retrospective study estimated clinically assigned hearing thresholds from incomplete ABR intensity series, using each ear as the prediction unit. We compared ABR-GraphDoseNet with three neural baselines and five ablation variants using ten-fold cross-validation grouped by subject. All recordings and both ears from a subject remained in the same fold, preventing subject-level overlap between training and test data. This design reduces the optimistic bias that can arise from splitting correlated records independently [30,31,32]. Internal validation subjects guided model selection, while held-out predictions supported assessment of error magnitude, tolerance-based accuracy, and threshold-specific errors.

### 2.2. Dataset and Data Acquisition

#### 2.2.1. Participants and ABR Recordings

This retrospective study analyzed auditory brainstem response (ABR) recordings acquired using the SmartEP system (Intelligent Hearing Systems, Miami, FL, USA) [33]. All examinations took place in an acoustically attenuated and electromagnetically shielded room. The initial cohort included 1149 subjects aged 0–17 years, comprising 713 males and 436 females, with a mean age of 2.26±2.82 years. Clinical assessments by experienced audiology professionals classified 432 subjects as having normal hearing and 717 as having varying degrees of hearing abnormality.

Click stimuli were presented separately to the left and right ears at nominal intensities from 10 to 100 dB nHL in steps of 10 dB, with a presentation rate of 19.1 stimuli/s. Each ABR recording contained up to 1024 stimulus repetitions, referred to as sweeps. During routine acquisition, recording at a given stimulus intensity could terminate early once a clear and repeatable response appeared. If no repeatable waveform appeared, the system stopped automatically after 1024 sweeps. The response at the preceding level generally guided selection of the next stimulus intensity. Consequently, the final stimulus intensity series for each ear was often incomplete.

A total of 9344 ABR waveforms from 2064 ears of 1045 subjects were included for threshold estimation. Each waveform was associated with a subject identifier, ear side, stimulus intensity, and clinically determined hearing threshold. Because hearing thresholds were defined at the ear level, all subsequent analyses treated each individual ear, rather than each waveform, as the prediction unit.

#### 2.2.2. Electrode Montage and Signal Acquisition Settings

Electrode placement followed the international 10–20 system. The active electrode was placed at Cz, the reference electrodes were positioned on the left and right mastoids at A1 and A2, respectively, and the ground electrode was placed on the forehead. The amplifier gain was set to 100,000. During acquisition, the system applied a high-pass filter at 100 Hz, a low-pass filter at 3000 Hz, and a mains frequency filter.

Artifact rejection used an amplitude threshold of ±31.0μV within a rejection window of 1.0–10.0 ms. Each original ABR waveform covered the interval from −13.675 to 11.900 ms relative to stimulus onset, contained 1024 sampling points, and was sampled at 40 kHz. The threshold estimation task used only the interval after stimulus onset that contained the principal clinically relevant ABR response.

#### 2.2.3. Clinical Labels and Ethical Approval

Qualified audiology professionals determined a hearing threshold for each ear during routine clinical assessment. The readers jointly examined the waveform series from the same ear across multiple stimulus intensities and assigned the lowest stimulus level at which a repeatable response remained detectable as the reference threshold. This clinically determined threshold served as the target label for the supervised learning task.

For recordings with a clearly identifiable Wave V, two experienced audiologists also annotated its peak and latency. These annotations provided auxiliary clinical waveform information rather than the primary prediction target for the ear. The clinically assigned thresholds defined categories of hearing loss severity for dataset description and stratified analyses.

This study involved a secondary analysis of existing research data obtained from the corresponding author of a previously published study upon request and with authorization. No new participants were recruited, and no additional human-subject data or biological samples were collected for the present study. Therefore, no additional institutional review board approval was required for this secondary analysis. The original data collection was conducted with approval from the relevant institutional ethics committee and in accordance with applicable ethical requirements.

### 2.3. Overview of ABR-GraphDoseNet

ABR-GraphDoseNet estimates the hearing threshold from the complete waveform series at multiple intensities for one ear. Rather than treating waveforms at different stimulus levels as independent samples, the model organizes all recordings from the same ear on a fixed intensity grid and represents them as a masked stimulus intensity response graph.

As illustrated in Figure 1, the overall framework consists of five stages. First, the pipeline crops each waveform, corrects its baseline, resamples it, and standardizes it. The normalized waveform and its first and second temporal differences form a representation with three channels. Second, the pipeline maps recordings from one ear to fixed stimulus intensity nodes, while a binary observation mask identifies the measured nodes.

Third, a temporal morphology tokenizer converts the waveform at each stimulus level into a compact node representation. A learnable stimulus intensity embedding preserves the physical stimulus intensity. Fourth, masked graph attention blocks exchange information across observed nodes through relationships between adjacent stimulus levels and adaptive relationships determined by waveform features.

Finally, two complementary prediction branches receive the node representations refined by the graph. A boundary decoder scores candidate threshold transitions between neighboring stimulus levels, whereas a global threshold head aggregates evidence from the entire observed graph. Joint optimization and score fusion produce the final hearing threshold estimate.

### 2.4. Waveform Preprocessing

For each ABR recording, we retained the waveform segment from 0 to 10 ms after stimulus onset. Let $x\in R^{T_{0}}$ denote the cropped waveform, where $T_{0}$ is the number of original samples in the selected time interval.

We corrected the baseline by subtracting the mean of the first 5% of samples from the complete waveform, then resampled the result to T=512 temporal points.

Each waveform was independently standardized using

(1) $$\tilde{x}=\frac{x-\mu_{x}}{\sigma_{x}+\epsilon },$$

where $\tilde{x}\in R^{T}$ is the normalized waveform, $\mu_{x}$ and $\sigma_{x}$ are the mean and standard deviation of the resampled waveform, respectively, and ϵ is a small positive constant used to avoid division by zero.

The first temporal difference, denoted by $\Delta \tilde{x}$, describes local slope changes. The second temporal difference, denoted by $\Delta^{2}\tilde{x}$, characterizes local changes in slope and waveform curvature.

We constructed the final waveform representation as

(2) $$X_{\mathrm{wav}}=\left[\tilde{x},\Delta \tilde{x},\Delta^{2}\tilde{x}\right]\in R^{T\times C},$$

where $X_{\mathrm{wav}}$ denotes the waveform input tensor, T=512 is the number of temporal points, and C=3 is the number of channels. The channels represent the normalized waveform and its first and second differences.

### 2.5. Stimulus Intensity Response Graph Construction for Each Ear

#### 2.5.1. Fixed Stimulus Intensity Grid

We mapped all recordings from the same ear to the following descending stimulus intensity grid:

(3) D={100,90,80,70,60,50,40,30,20,10,0}dBnHL.

Here, D denotes the set of nominal stimulus intensities. The grid contained K=11 nodes, and the *i*th node corresponded to the nominal stimulus intensity $d_{i}$. The 0 dB nHL node was a virtual lower anchor, not an acquired stimulus level; it allowed the model to represent the 10 dB threshold boundary and remained unobserved because the acquisition protocol started at 10 dB nHL.

We assigned a waveform acquired at an actual intensity $d^{\mathrm{obs}}$ to the nearest nominal node if the absolute difference between $d^{\mathrm{obs}}$ and $d_{i}$ did not exceed 5.1 dB. If multiple recordings mapped to the same node, we averaged their preprocessed waveform tensors to obtain one representation for that node.

This fixed design ensured that the same node index represented the same physical intensity across all ears, even when the measured combinations differed.

#### 2.5.2. Observation Mask

Because the clinical intensity series were incomplete, we constructed a binary observation mask:

(4) $$m_{i}=\left\{\begin{array}{cc} 1, & \mathrm{if}\;a\;\mathrm{waveform}\;\mathrm{was}\;\mathrm{observed}\;\mathrm{at}\;\mathrm{node}\;i,\\ 0, & \mathrm{otherwise}. \end{array}\right.$$

Here, $m_{i}$ denotes the observation state of the *i*th stimulus node. The complete mask is denoted by $m={\left[m_{1},\ldots ,m_{K}\right]}^{⊤}\in {\left\{0,1\right\}}^{K}$.

For an unmeasured node, zeros fill the corresponding waveform tensor only to maintain a fixed input size. The observation mask remained active throughout graph reasoning and global pooling, which prevented the model from interpreting padding as a measured waveform without a physiological response.

We represent the complete input for one ear as

(5) $$G=\left(X_{\mathrm{ear}},d,m\right),$$

where G denotes the masked stimulus intensity response graph of one ear, $X_{\mathrm{ear}}\in R^{K\times T\times C}$ is the waveform tensor across intensities, $d={\left[d_{1},\ldots ,d_{K}\right]}^{⊤}$ is the ordered intensity vector, and m is the observation mask.

### 2.6. Temporal Morphology Tokenizer

For node *i*, the corresponding waveform input is denoted by $X_{i}\in R^{T\times C}$. A temporal morphology tokenizer converts this waveform tensor into a compact node token.

The tokenizer contained two depthwise separable one-dimensional convolutional layers with kernel sizes of 11 and 7. A Gaussian error linear unit activation, layer normalization, and dropout followed each layer. A pointwise convolution then integrated information across the waveform and temporal difference channels.

Let $U_{i}\in R^{T^{\prime }\times d}$ denote the resulting temporal feature sequence, where $T^{\prime }$ is the temporal length after convolutional processing and d=96 is the feature dimension. The feature vector at temporal position *t* is denoted by $u_{it}\in R^{d}$.

A learnable attention pooling operation produces one morphology token:

(6) $$h_{i}=\sum_{t=1}^{T^{\prime }}\alpha_{it}u_{it},$$

where $h_{i}\in R^{d}$ is the morphology token of node *i*, and $\alpha_{it}$ is the normalized attention weight assigned to temporal feature $u_{it}$. The attention weights are nonnegative and sum to one across all $T^{\prime }$ temporal positions.

This operation allows the model to emphasize temporally localized waveform structures instead of assigning equal importance to all samples.

### 2.7. Graph Reasoning with Stimulus Intensity Information

#### 2.7.1. Stimulus Intensity Embedding

The same waveform morphology may have different clinical meanings at different stimulus intensities. Therefore, the model maps each nominal intensity $d_{i}$ to a learnable stimulus intensity embedding $e_{i}\in R^{d}$.

We define the initial node representation as

(7) $$v_{i}^{\left(0\right)}=h_{i}+e_{i},$$

where $v_{i}^{\left(0\right)}\in R^{d}$ is the initial representation of node *i*, $h_{i}$ is its waveform morphology token, and $e_{i}$ is the corresponding stimulus intensity embedding. The superscript (0) denotes the input to the first graph reasoning block.

#### 2.7.2. Masked Graph Attention

The graph reasoning module used attention with multiple heads to exchange information among observed stimulus nodes. For each head, the projected node representations determined the similarity between query node *i* and key node *j*. The attention score also included two terms:

(8) $$A_{ij}=\frac{q_{i}^{⊤}k_{j}}{\sqrt{d_{r}}}+b_{ij}^{\mathrm{adj}}+b_{ij}^{\mathrm{adapt}},$$

where $A_{ij}$ is the unnormalized attention score from node *j* to node *i*, $q_{i}\in R^{d_{r}}$ is the query vector of node *i*, $k_{j}\in R^{d_{r}}$ is the key vector of node *j*, and $d_{r}$ is the feature dimension of one attention head.

The term $b_{ij}^{\mathrm{adj}}$ denotes the learnable bias between adjacent stimulus levels. It describes the structural relationship between neighboring stimulus levels and preserves intensity order. The term $b_{ij}^{\mathrm{adapt}}$ denotes the adaptive edge bias, which the model generates from pairwise waveform features of nodes *i* and *j*. This term allows informative interactions between nonadjacent stimulus levels.

The model applied the observation mask before attention normalization. Thus, a node with $m_{j}=0$ could not contribute key or value information during graph message passing. Residual connections, layer normalization, dropout, and a feedforward network processed the concatenated outputs from all heads.

The final model contained two graph reasoning blocks with four attention heads. The symbol $z_{i}\in R^{d}$ denotes the representation of node *i* after graph reasoning.

### 2.8. Decoder for Adjacent Boundaries

The K=11 stimulus intensity nodes form K−1=10 adjacent node pairs. Each pair corresponds to one candidate threshold boundary.

For the boundary between nodes *i* and i+1, we construct the boundary feature as

(9) $$p_{i}=\left[z_{i}∥z_{i+1}∥|z_{i}-z_{i+1}|∥\left(z_{i}⊙z_{i+1}\right)∥m_{i}∥m_{i+1}\right],$$

where $p_{i}$ is the feature vector of the *i*th candidate boundary, $z_{i}$ and $z_{i+1}$ are the representations of the two adjacent nodes after graph reasoning, ‖ denotes vector concatenation, |·| denotes absolute difference by element, and ⊙ denotes multiplication by element. The variables $m_{i}$ and $m_{i+1}$ indicate whether the two nodes were observed.

The feature vector, therefore, contains the two node states, their difference, their interaction, and their observation states. A multilayer perceptron mapped $p_{i}$ to a scalar boundary logit $o_{i}^{\mathrm{bd}}$. The complete vector of boundary logits is denoted by

(10) $$o^{\mathrm{bd}}={\left[o_{1}^{\mathrm{bd}},\ldots ,o_{K-1}^{\mathrm{bd}}\right]}^{⊤},$$

where each element represents the unnormalized score of one candidate threshold boundary.

### 2.9. Global Threshold Head

A candidate threshold boundary may be difficult to identify when the adjacent waveforms are weak, noisy, or missing. A global threshold head was, therefore, introduced to aggregate evidence from all observed stimulus nodes.

A learnable scalar function assigned one pooling score to each node after graph reasoning. We calculated the masked attention pooling weight as

(11) $$\beta_{i}=\frac{m_{i}\exp\left(s\left(z_{i}\right)\right)}{\sum_{j=1}^{K}m_{j}\exp\left(s\left(z_{j}\right)\right)},$$

where $\beta_{i}$ is the pooling weight of node *i*, s(·) is a learnable scalar scoring function, and *j* is the summation index over all *K* nodes. The observation indicator $m_{i}$ ensures that only measured nodes receive nonzero pooling weights.

The global representation for the ear was

(12) $$g_{\mathrm{ear}}=\sum_{i=1}^{K}\beta_{i}z_{i},$$

where $g_{\mathrm{ear}}\in R^{d}$ summarizes the waveform evidence distributed across the observed stimulus levels.

The model also transformed the observation mask m into a learnable representation ϕ(m). A threshold classifier received the concatenated global ear representation and mask representation, then generated the direct vector of threshold logits $o^{\mathrm{th}}\in R^{K-1}$.

### 2.10. Joint Optimization and Threshold Prediction

The training procedure jointly optimized the boundary decoder and global threshold head. Let y∈{1,…,K−1} denote the reference threshold class. Both output branches used the same ordered space of threshold classes.

The total training loss was

(13) $$L=L_{\mathrm{bd}}+\lambda L_{\mathrm{th}},$$

where $L_{\mathrm{bd}}$ is the cross-entropy loss of the branch for adjacent boundaries, $L_{\mathrm{th}}$ is the cross-entropy loss of the global threshold branch, and λ=0.3 controls the contribution of the global threshold objective.

During inference, we fused the two output vectors as

(14) $$o^{\mathrm{fuse}}=o^{\mathrm{bd}}+\gamma o^{\mathrm{th}},$$

where $o^{\mathrm{fuse}}\in R^{K-1}$ is the fused logit vector and γ is the fusion coefficient selected using only the internal validation set.

The class with the highest fused logit became the prediction. Its index then mapped to the corresponding hearing threshold intensity in dB nHL.

### 2.11. Experimental Platform and Model Training

We conducted the experiments using Python 3.8 and TensorFlow 2.9.0 under Ubuntu 20.04 with CUDA 11.2. We trained the models on one NVIDIA RTX A6000 GPU with 48 GB of memory. The computing platform also included a 16-core Intel Xeon Platinum 8352V CPU operating at 2.10 GHz and 120 GB of system memory.

Hyperparameter selection combined grid search and random search within empirically defined ranges. Grid search evaluated the learning rate, token dimension, number of graph reasoning blocks, and number of attention heads. Random search explored dropout, regularization, and prediction fusion settings.

Only the internal validation set, which contained separate subjects, guided evaluation of candidate configurations. Validation performance controlled early stopping to reduce unnecessary computation and limit overfitting. The test set did not contribute to hyperparameter selection, model selection, early stopping, or selection of the fusion coefficient.

### 2.12. Comparison Methods

We compared ABR-GraphDoseNet with three neural architectures that represent progressively broader context: a one-dimensional convolutional neural network (1D CNN), a bidirectional long short-term memory network (BiLSTM), and a Transformer. All baselines used the same preprocessed waveform series, fixed stimulus intensity grid, observation mask, threshold classes, and data partitions as the proposed model.

The 1D CNN extracted local temporal morphology from each waveform and pooled the resulting features across the available stimulus levels. BiLSTM processed the intensity level features in intensity order to model sequential dependence in both directions. Transformer used self-attention to integrate information across the complete observed intensity series. Each baseline produced a direct distribution over the threshold classes. We selected its hyperparameters using only the internal validation data and evaluated it with the same ten-fold protocol grouped by subject as ABR-GraphDoseNet. This design isolates the effect of the model structure while holding the input representation and evaluation procedure constant.

### 2.13. Ablation Experiments

We conducted ablation experiments to evaluate the principal components of ABR-GraphDoseNet. The variants included:

- removal of the adaptive edge bias while retaining the ordered relationships between adjacent stimulus levels;
- removal of the explicit boundary decoder;
- replacement of the temporal morphology tokenizer with a simpler convolutional encoder;
- replacement of graph attention with a graph convolutional network that used fixed adjacent stimulus levels;
- removal of the global threshold head.

All ablation variants used the same preprocessing procedure, data partitions grouped by subject, training protocol, and evaluation metrics as the complete model.

We additionally examined observation-pattern confounding using the same subject-grouped ten-fold evaluation protocol and the same 2064 ears. An observation-mask-only baseline received indicators of measured stimulus intensities without ABR waveforms. A second variant retained waveform-based inputs but removed explicit mask encoding. We compared both with the complete model. Removing explicit mask encoding does not eliminate implicit missingness information from the availability of intensity-specific inputs.

### 2.14. Statistical Analysis

We used complementary descriptive metrics because threshold errors differ in magnitude and clinical interpretation. MAE summarizes absolute error in dB, whereas RMSE gives greater weight to large errors. Exact accuracy measures agreement with the assigned threshold class; the primary endpoint, Acc@10, measures agreement within one 10 dB intensity step without implying that every such error is clinically acceptable. Main and component-ablation results are reported as mean ± standard deviation across ten subject-grouped folds; the preliminary observation-pattern comparison reports MAE and Acc@10 point estimates. These standard deviations describe variation across held-out partitions, not confidence intervals or formal statistical significance; comparisons are descriptive.

We evaluated model performance for each ear. Let *N* denote the number of evaluated ears, $\tau_{n}$ denote the clinically assigned threshold of the *n*th ear, and ${\hat{\tau }}_{n}$ denote its predicted threshold.

We calculated the mean absolute error as

(15) $$\mathrm{MAE}=\frac{1}{N}\sum_{n=1}^{N}\left|{\hat{\tau }}_{n}-\tau_{n}\right|,$$

where *n* indexes the evaluated ears.

We calculated the root mean squared error as

(16) $$\mathrm{RMSE}=\sqrt{\frac{1}{N}\sum_{n=1}^{N}{\left({\hat{\tau }}_{n}-\tau_{n}\right)}^{2}}.$$

Exact accuracy equals the proportion of ears for which the predicted threshold matched the reference threshold. We define accuracy within 10 dB, denoted by Acc@10, as the proportion of ears satisfying

(17) $$|{\hat{\tau }}_{n}-\tau_{n}|\leq 10\;\mathrm{dB}.$$

Because the output thresholds followed a grid with steps of 10 dB, an error of one threshold class corresponded to a difference of 10 dB. In addition to summary metrics for each fold, pooled held-out predictions supported analysis by threshold, error direction, severe errors, and representative cases.

## 3. Results

Figure 2 shows threshold-class agreement, Table 1 and Figure 3 compare the baseline models, and Table 2 and Figure 4 quantify the effects of component ablation.

### 3.1. Overview of Prediction Performance

Under the ten-fold evaluation grouped by subject, ABR-GraphDoseNet achieved an MAE of 0.896 ± 0.272 dB, an RMSE of 4.332 ± 1.256 dB, an exact accuracy of 93.80 ± 1.41%, and a primary endpoint Acc@10 of 98.64 ± 1.01%. These fold summaries indicate close agreement with recorded thresholds for subjects excluded from model training.

Figure 2 resolves that aggregate agreement by threshold class. The dominant diagonal shows that the model reproduced most recorded labels exactly, while the sparse off-diagonal entries occur mainly at adjacent levels. The error structure, therefore, supports the tolerance result without repeating the fold metrics: most disagreements reflect one step on the clinical intensity grid rather than broad displacement across classes.

Together, the fold summaries and class-level distribution show stable internal reproduction of recorded ear thresholds. They do not establish agreement with an independent physiological reference standard or performance under an external acquisition protocol.

### 3.2. Comparison with Deep Learning Baselines

The baselines represent local convolution with pooling (1D CNN), bidirectional sequence modeling (BiLSTM), and global self-attention (Transformer), whereas ABR-GraphDoseNet explicitly models intensity relationships and combines boundary-level and series-level evidence.

Table 1 reports the exact fold summaries, while Figure 3 shows the cross-model ordering and fold variation. Performance improved from 1D CNN to BiLSTM and then Transformer, which indicates that integrating context across stimulus levels benefits this task. ABR-GraphDoseNet retained the most favorable distribution for both error and accuracy criteria, so its advantage did not depend on a single metric.

Transformer was the strongest baseline. Relative to it, ABR-GraphDoseNet reduced MAE by 0.417 dB and RMSE by 1.603 dB, while exact accuracy and Acc@10 increased by 1.41 and 1.06 percentage points. This residual gain after global self-attention suggests that the explicit intensity graph and the separation of local boundary evidence from global ear evidence contribute beyond generic sequence integration.

### 3.3. Ablation Results

Table 2 provides the exact values for each model variant, and Figure 4 makes the relative sensitivity of the components visible. Every tested modification shifted at least one error or accuracy measure in an unfavorable direction, but the magnitude varied substantially across components.

Removing the global threshold head caused the largest deterioration and more than doubled MAE, which identifies aggregation across the full ear series as the dominant tested contributor. Replacing the morphology tokenizer or graph attention produced the next clearest losses. Removing adaptive edges or replacing the boundary decoder had smaller effects, which indicates complementary value rather than a dominant source of accuracy.

The ablation pattern, therefore, supports a layered interpretation of the model. Waveform morphology and global aggregation form the main predictive backbone, while graph interaction and boundary structure refine that evidence. The complete model achieved the strongest joint result, but the results do not justify attributing the gain to adaptive edges alone.

### 3.4. Model Interpretation and Visualization

Figure 5 examines where ABR-GraphDoseNet placed normalized decision importance across stimulus levels. At the population level, Figure 5a shows a band of high importance near the recorded threshold region, while unobserved combinations remain gray. The concentration follows the threshold axis rather than one fixed stimulus level. This pattern is inconsistent with reliance on a single universal level, but it does not exclude other shortcuts related to acquisition.

Figure 5b connects this population pattern to individual ears. In the three correct examples, the largest importance aligns with the waveform at the recorded and predicted threshold. In the error example, importance splits between the true 10 dB level and the incorrectly predicted 60 dB level, with greater weight at 60 dB. The visualization, therefore, reveals both the intended use of threshold-relevant evidence and a concrete failure mode in which a suprathreshold waveform dominates the decision.

These patterns make the decision process auditable at the stimulus level, but they do not prove physiological validity or causal use of waveform morphology. They instead identify which recorded levels deserve closer review in a concordant or discordant case.

### 3.5. Observation-Pattern Confounding Analysis

The observation-mask-only baseline achieved an MAE of 1.978 dB and an Acc@10 of 94.95% (Table 3). The model without explicit mask encoding achieved 1.541 dB and 96.51%, respectively, while the complete model achieved 0.896 dB and 98.64%. These preliminary results show that observation patterns carry threshold-related information, but do not alone account for the complete model’s performance. The comparison does not isolate a waveform-specific effect because missingness remains implicitly available in the waveform-based inputs.

## 4. Discussion

This study investigated hearing threshold estimation from incomplete ABR recordings at multiple intensities using ABR-GraphDoseNet. Three findings define the scope of the evidence. First, ABR-GraphDoseNet achieved the strongest overall performance under validation with subjects grouped across ten folds, outperforming representative convolutional, recurrent, and attention models across MAE, RMSE, exact accuracy, and Acc@10. Second, the global threshold head and morphology tokenizer produced the largest performance changes when removed or replaced. Third, graph interaction, adaptive connections across intensities, and explicit boundary modeling provided smaller improvements beyond conventional sequence modeling. Together, these findings support structured stimulus intensity response modeling for each ear rather than treating the series as independent waveforms or a generic temporal sequence.

### 4.1. Stimulus Intensity Response Modeling Improves Threshold Estimation Across Intensities

ABR-GraphDoseNet achieved an MAE of 0.896 dB and an Acc@10 of 98.64%, compared with 1.313 dB and 97.58% for Transformer, the strongest baseline. The progression from 1D CNN through BiLSTM to Transformer is consistent with the value of integrating information across intensities. The further improvement with ABR-GraphDoseNet supports explicit intensity relationships within the tested dataset, but does not establish their superiority across acquisition settings.

Earlier studies also demonstrate effective threshold estimation without an explicit intensity graph. ABRpresto uses resampled cross-correlation across subaverages [24], while Gaussian-process methods estimate response amplitude across levels and use active learning for stimulus selection [10]. These findings limit any claim that graph modeling is necessary for automated threshold estimation. Their inputs, acquisition objectives, and validation settings differ from our retrospective prediction of clinical labels, so published results do not establish a direct performance ranking. Our comparisons support gains over the three tested neural baselines, not superiority over all existing ABR methods or prospective acquisition strategies.

This distinction matters because responses at different stimulus intensities form an ordered pattern in which waveform morphology changes as intensity approaches the hearing threshold. Conventional sequence models can learn dependencies across inputs, but they do not explicitly encode the stimulus intensity response structure of the examination. In ABR-GraphDoseNet, each stimulus intensity has a defined graph position, so the model interprets waveform information jointly with the associated stimulus level and its relation to other measured intensities.

The representation also accommodates incomplete intensity series, which are common in retrospective ABR examinations. ABR-GraphDoseNet retains the nominal intensity structure while recording whether each level was observed, so it can integrate available measurements without interpolating missing waveforms or equating missing levels with measured responses. The present internal performance, therefore, supports structured stimulus intensity response modeling as a useful strategy for incomplete ABR series, subject to external validation.

### 4.2. Global Evidence and Waveform Morphology Are Major Contributors

The ablation experiments clarify which components contribute most strongly to ABR-GraphDoseNet. Removal of the global threshold head produced the largest degradation, increasing MAE from 0.896 to 2.156 dB and reducing Acc@10 from 98.64% to 95.69%. This change indicates that hearing threshold estimation benefits strongly from integrating information across all available stimulus intensities.

This finding is consistent with clinical ABR assessment, where one waveform rarely determines the threshold in isolation. Interpretation instead depends on whether characteristic morphology persists, attenuates, or disappears as intensity decreases. The global threshold head aggregates this distributed evidence across the complete ear series and reduces dependence on any single local transition.

The morphology tokenizer represented another important component. Replacing it with a conventional CNN encoder increased MAE from 0.896 to 1.570 dB and reduced exact accuracy from 93.80% to 90.99%. The result indicates that explicit morphology representation before integration across intensities contributes information beyond generic convolutional encoding. It does not, however, isolate which temporal feature accounts for that contribution.

Together, these two ablations identify local waveform characterization and global integration across stimulus intensities as the principal predictive backbone of ABR-GraphDoseNet. Morphology encoding determines what information the model extracts from each response, whereas the global threshold head determines how it combines that evidence for the ear.

### 4.3. Graph Interaction Provides Complementary Information Across Intensities

The graph ablations indicate that information exchange across stimulus intensities also contributes to performance. Replacing graph attention with a conventional GCN increased MAE from 0.896 to 1.467 dB and reduced Acc@10 from 98.64% to 96.85%. Under the tested configurations, adaptive weighting of information from different stimulus levels, therefore, outperformed the more constrained GCN aggregation.

Removing adaptive edges resulted in a smaller performance reduction, with MAE increasing to 1.294 dB and Acc@10 decreasing to 97.48%. Relative to removal of the global threshold head, this change places adaptive edges in a complementary rather than dominant role. The result suggests that relationships beyond fixed neighboring intensities can provide useful information within this architecture.

This property may matter when the intensity series is incomplete. If a neighboring level is unavailable, information from observed but nonadjacent intensities may still contribute to the overall stimulus intensity response estimate. Adaptive graph interactions can integrate such information without assuming that all ears contain the same measurements.

Taken together, the GAT and adaptive edge ablations show that node representation alone does not explain the benefit of graph modeling. Information propagation among nodes also matters. Physical intensity order provides the structural foundation, while adaptive interaction refines the contribution of each measured level according to its waveform representation and context.

### 4.4. Local Boundary and Global Threshold Decisions Are Complementary

The boundary decoder provides additional structure by formulating hearing threshold estimation through transitions between neighboring stimulus levels. Replacing it with direct threshold classification increased MAE from 0.896 to 1.143 dB and reduced exact accuracy from 93.80% to 92.49%. This effect was smaller than the changes caused by removing the global threshold head or replacing the morphology tokenizer.

This finding suggests that explicit transition modeling complements global threshold classification. Hearing threshold estimation requires identifying where the response pattern changes as stimulus intensity varies, so candidate transitions between neighboring levels encode the ordered search process directly.

At the same time, the larger effect of removing the global threshold head indicates that local transition evidence alone is insufficient. In incomplete series, the levels immediately surrounding the final threshold may be unavailable, so a decision may require more distant suprathreshold responses or responses near the threshold. ABR-GraphDoseNet addresses this issue by combining local boundary information with global evidence from the ear series.

The ablation pattern, therefore, supports a complementary local and global interpretation. Boundary modeling represents candidate transitions, whereas global threshold modeling integrates the complete available intensity series. Their joint use produced the strongest overall performance.

### 4.5. Structured Modeling by Ear Is the Main Methodological Contribution

Beyond numerical improvement over the evaluated baselines, the main methodological contribution lies in reformulating ABR threshold estimation as structured prediction for each ear. Most conventional waveform models focus on individual recordings or represent a set of recordings as a generic sequence. In contrast, ABR-GraphDoseNet associates each waveform with its physical stimulus intensity and integrates all available responses within one stimulus intensity response representation.

This formulation has three advantages. It preserves intensity order, represents missing levels explicitly, and separates waveform morphology, interaction across intensities, local transition modeling, and global threshold estimation into identifiable components. The ablation experiments can, therefore, test how these sources of information contribute to the prediction.

The comparison with 1D CNN, BiLSTM, and Transformer is informative in this context. These models progressively introduce local feature extraction, recurrent sequence modeling, and global attention, yet ABR-GraphDoseNet remained superior across all four metrics. The improvement, therefore, cannot be explained only by adding broader context to a generic neural sequence model. Structure tied to physical intensity provides additional predictive value in this dataset.

Importantly, the present findings should be interpreted as support for the complete structured framework rather than as evidence that any individual graph mechanism is universally superior to alternative neural architectures. The ablation experiments identify useful components within ABR-GraphDoseNet, but broader comparisons under different datasets, acquisition protocols, and model configurations will be required to determine how generally these advantages persist.

### 4.6. Implications for Automated ABR Assessment

The high agreement achieved by ABR-GraphDoseNet suggests potential value for computer support in hearing threshold estimation. Manual interpretation requires clinicians to integrate waveform morphology across several stimulus levels, which can be time-consuming when recordings are incomplete, noisy, or ambiguous near the threshold. A model that integrates the complete response pattern for an ear could provide an additional quantitative estimate.

A realistic initial application is clinical decision support rather than autonomous diagnosis. ABR-GraphDoseNet could provide an independent threshold estimate after the conventional recording procedure, allowing clinicians to compare model output with their interpretation. Cases with substantial disagreement could then receive additional review. Such a workflow would preserve clinical oversight while potentially improving consistency.

The current retrospective results support threshold estimation from recorded ABR series, but do not establish benefits for clinical decision-making or acquisition workflows. Whether model uncertainty or accumulated stimulus intensity response evidence can guide subsequent stimulus selection requires prospective evaluation.

### 4.7. Limitations and Future Validation

Two main limitations constrain interpretation. First, the study used retrospective data from a single source. Subject-grouped cross-validation prevented subject-level overlap but does not establish generalizability across devices, workflows, stimulation protocols, or intensity scaling and calibration.

Second, the missing stimulus levels in retrospective ABR examinations may not occur completely at random. The choice of tested intensities and the termination of the examination can be influenced by the examiner’s evolving assessment of the response. Consequently, the observation pattern itself may contain information related to the acquisition process in addition to the underlying physiological response. Our additional ten-fold analysis confirms that observation patterns contain threshold-related information: the mask-only baseline achieved an Acc@10 of 94.95%. The complete model outperformed this baseline, indicating predictive information beyond the observation pattern alone. However, removing explicit mask encoding does not yield a strictly waveform-only model because input availability can still reveal missingness. These results identify observation patterns as a potential confound without fully separating their contribution from waveform content. Controlled evaluation on ears with common intensity coverage, using identical observation patterns or artificial masking independent of threshold labels, remains necessary to isolate waveform-specific information.

External validation across centers, devices, populations, and clinical protocols is the primary next step, alongside controlled experiments separating waveform information from acquisition-related observation patterns.

## 5. Conclusions

This study proposed ABR-GraphDoseNet, an ear-level stimulus intensity response graph framework for hearing threshold estimation from multi-intensity ABR recordings, and demonstrated the effectiveness of jointly modeling information across stimulus levels. ABR-GraphDoseNet achieved the best overall performance under subject-grouped validation, indicating that integrating waveform morphology, stimulus intensity relationships, and global information can improve the accuracy and robustness of threshold prediction compared with treating recordings at different intensities independently. The ablation results further showed that the performance gain arose from the complementary contributions of multiple components rather than from a single module, with global threshold modeling and morphology representation providing the most substantial benefits, while boundary information and cross-intensity interactions offered additional improvements. These findings support structured ear-level modeling as an effective strategy for extracting threshold-related information from incomplete multi-intensity ABR series. Overall, ABR-GraphDoseNet provides a promising framework for automated ABR threshold estimation with strong predictive performance and interpretable decision structure, and offers a foundation for future studies on cross-center validation, clinical decision support, and more intelligent ABR assessment systems.

## Figures and Tables

**Figure 1:**
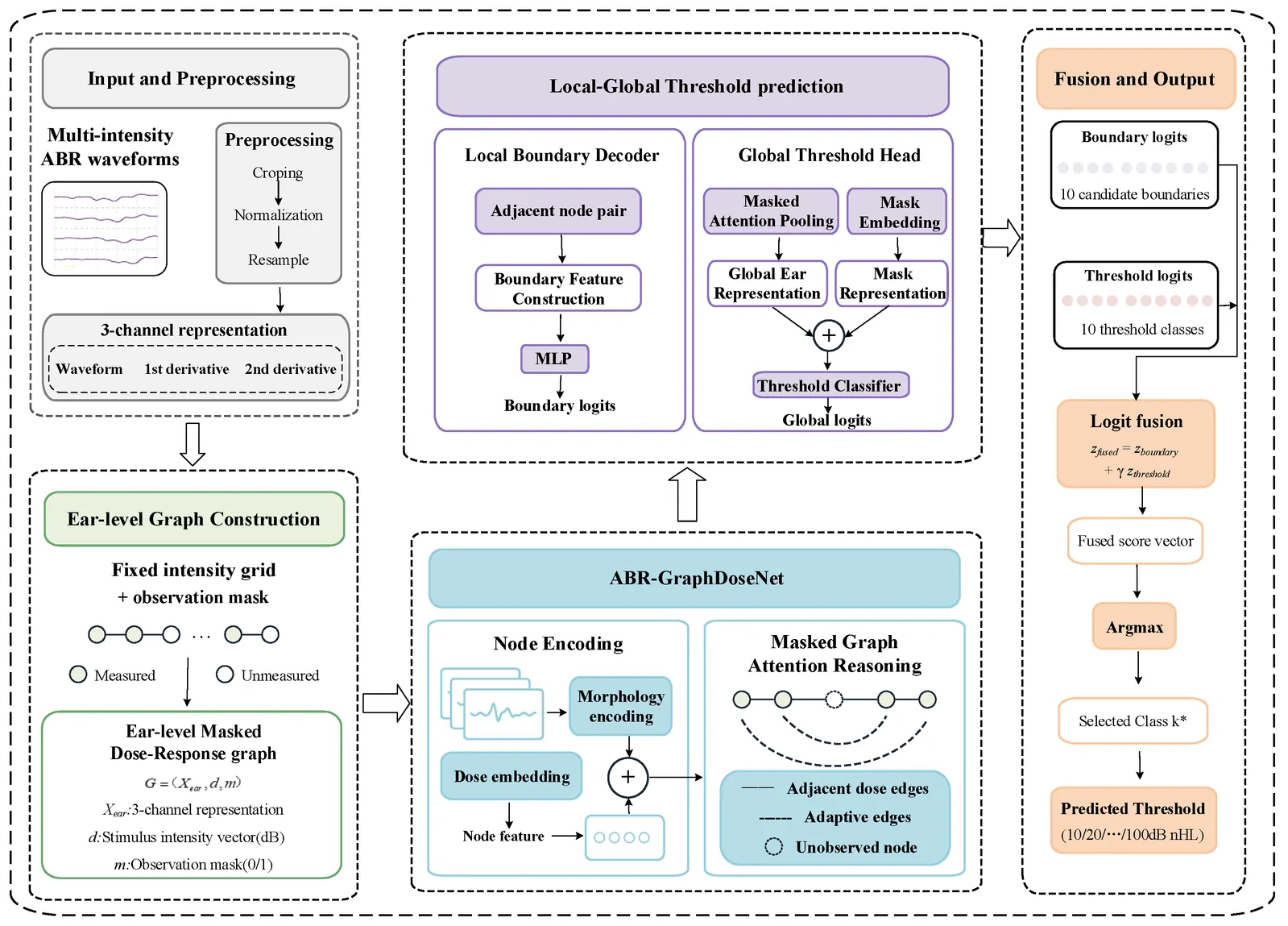
Overview of ABR-GraphDoseNet. Preprocessed waveforms and their first and second temporal differences form three-channel inputs on a fixed stimulus intensity grid, with an observation mask identifying measured levels. Morphology encoding and intensity embeddings feed masked graph attention with adjacent and adaptive edges. A local boundary decoder and a global threshold head provide complementary scores, which are fused to predict the hearing threshold for each ear.

**Figure 2:**
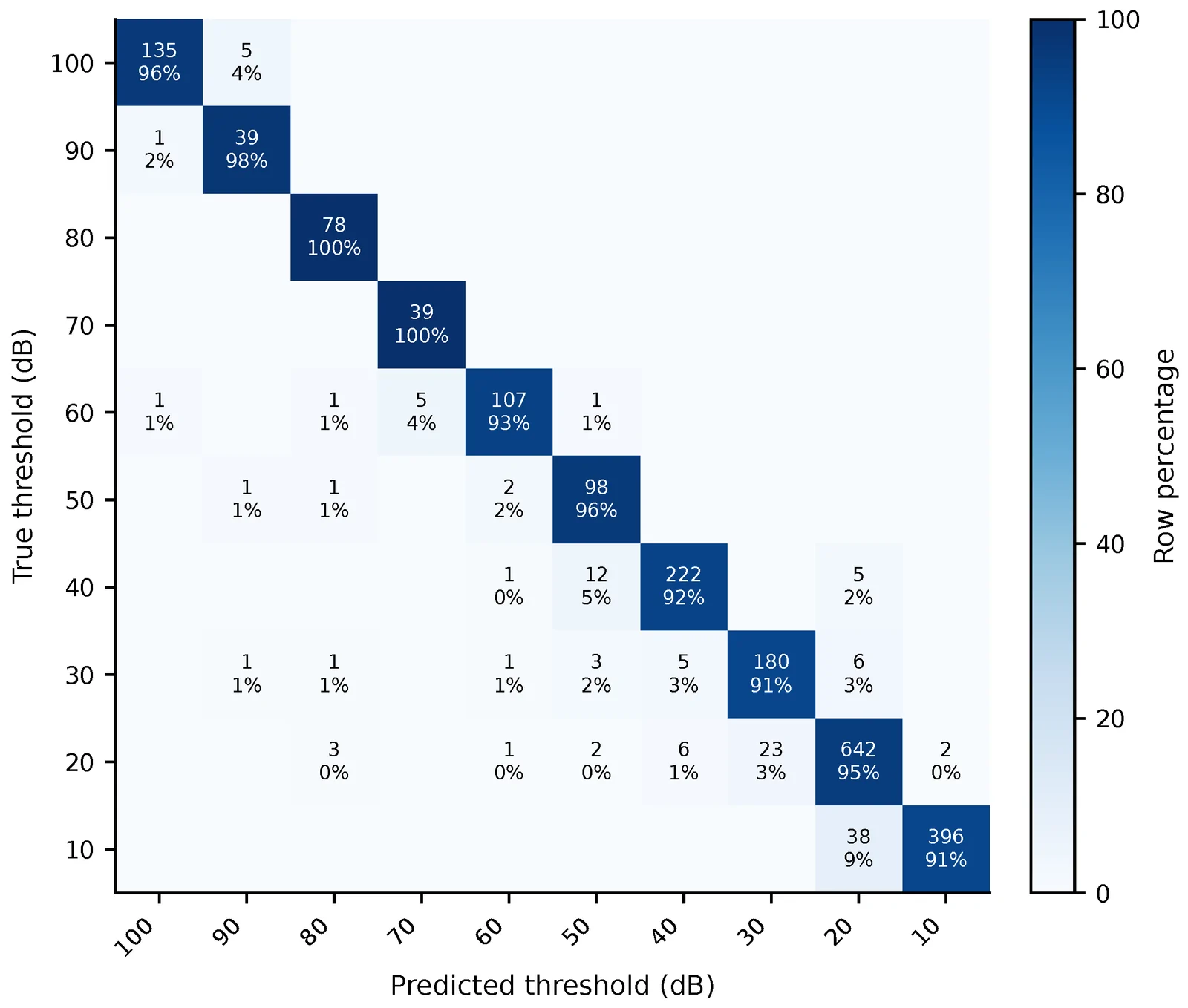
Confusion matrix pooling held-out predictions across ten folds grouped by subject for ABR-GraphDoseNet. Rows indicate clinically assigned thresholds and columns indicate predicted thresholds, both in dB nHL. Color encodes the row percentage; each populated cell reports the ear count and rounded percentage within its reference threshold class. Diagonal cells indicate exact agreement, whereas off-diagonal cells indicate prediction errors.

**Figure 3:**
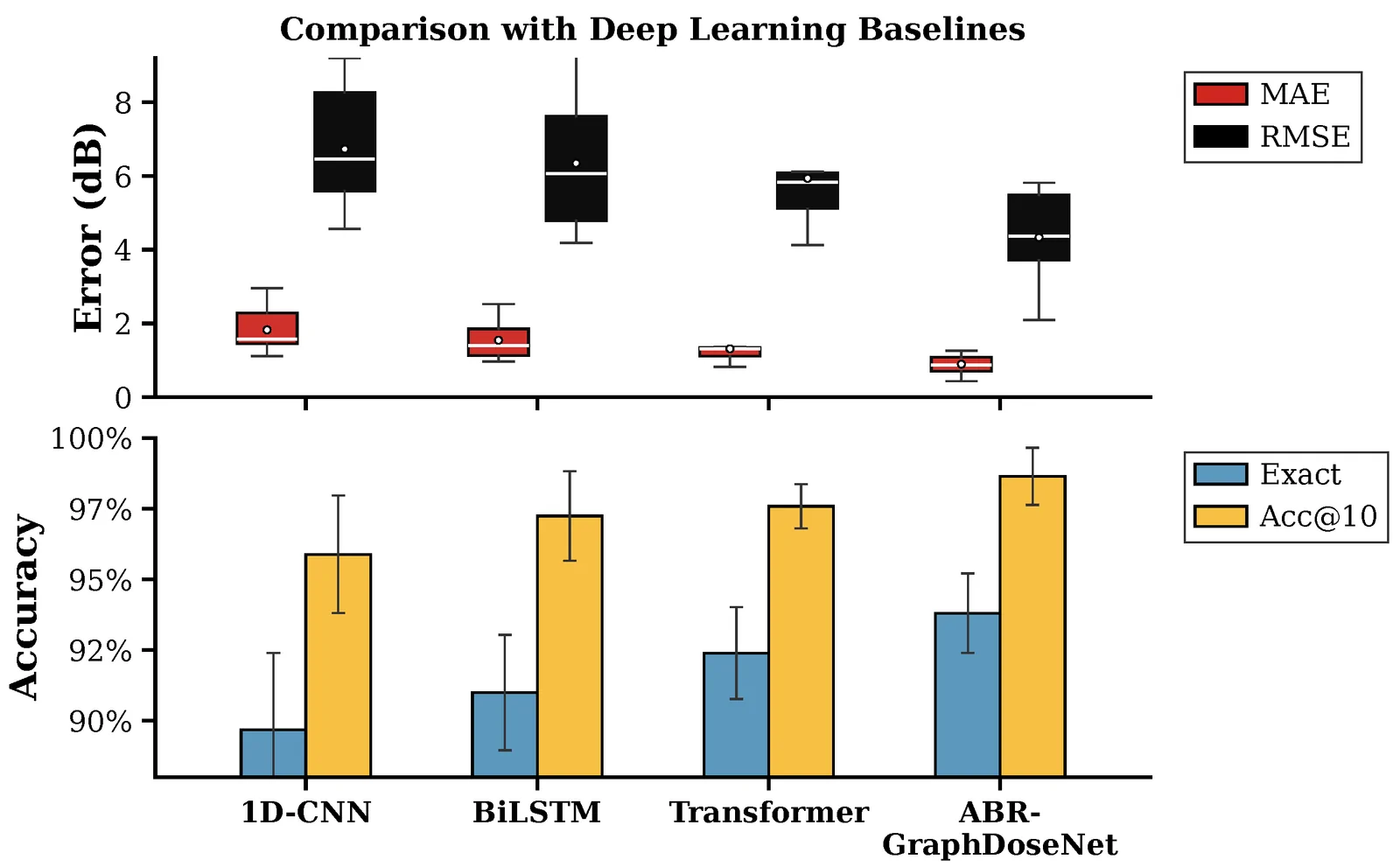
Comparison with deep learning baselines across ten folds grouped by subject. The (**upper panel**) summarizes MAE and RMSE distributions; the (**lower panel**) shows exact accuracy and Acc@10 with fold variation.

**Figure 4:**
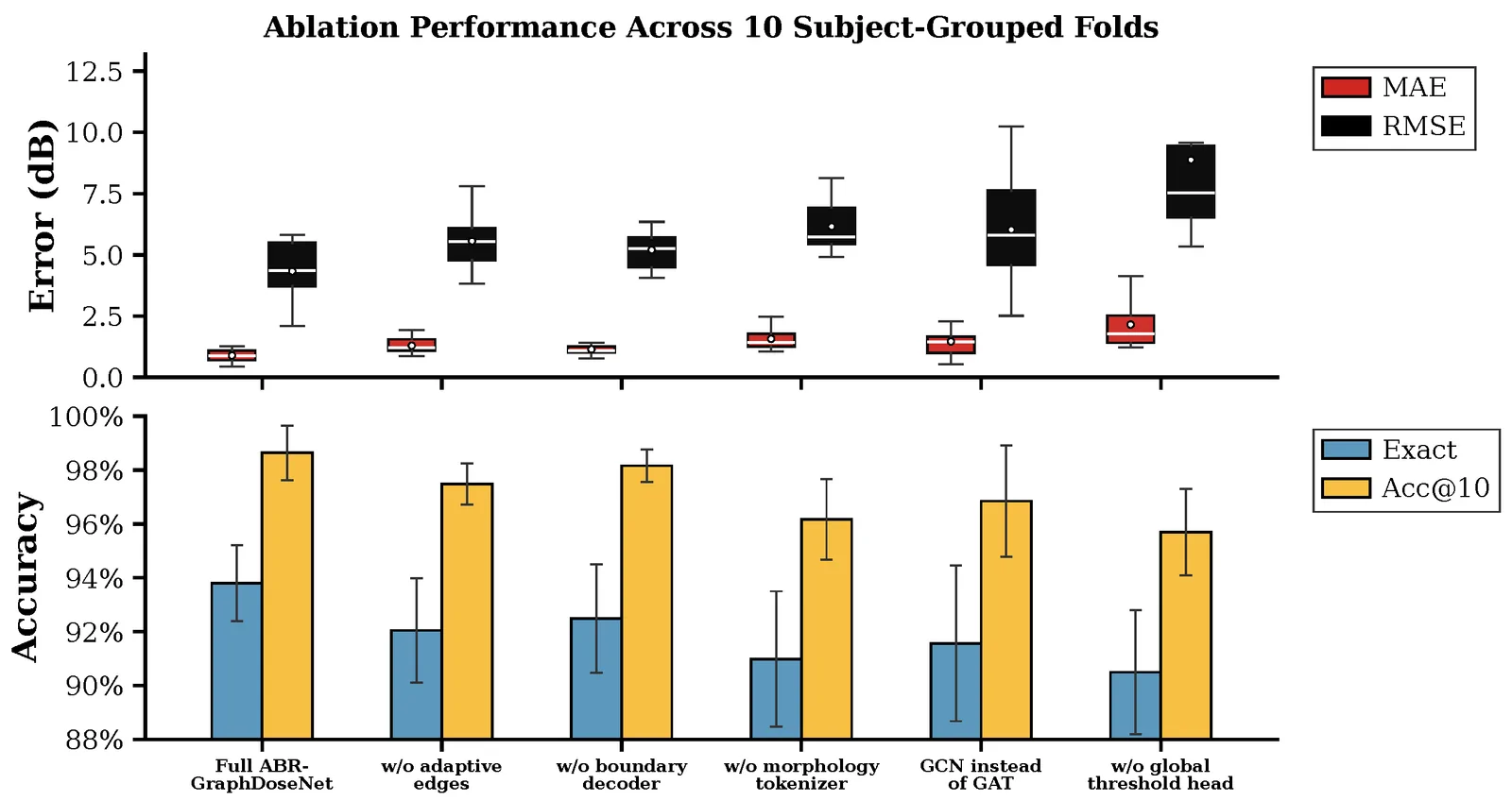
Ablation performance across ten folds grouped by subject. The (**upper panel**) summarizes MAE and RMSE distributions; the (**lower panel**) shows exact accuracy and Acc@10 with fold variation.

**Figure 5:**
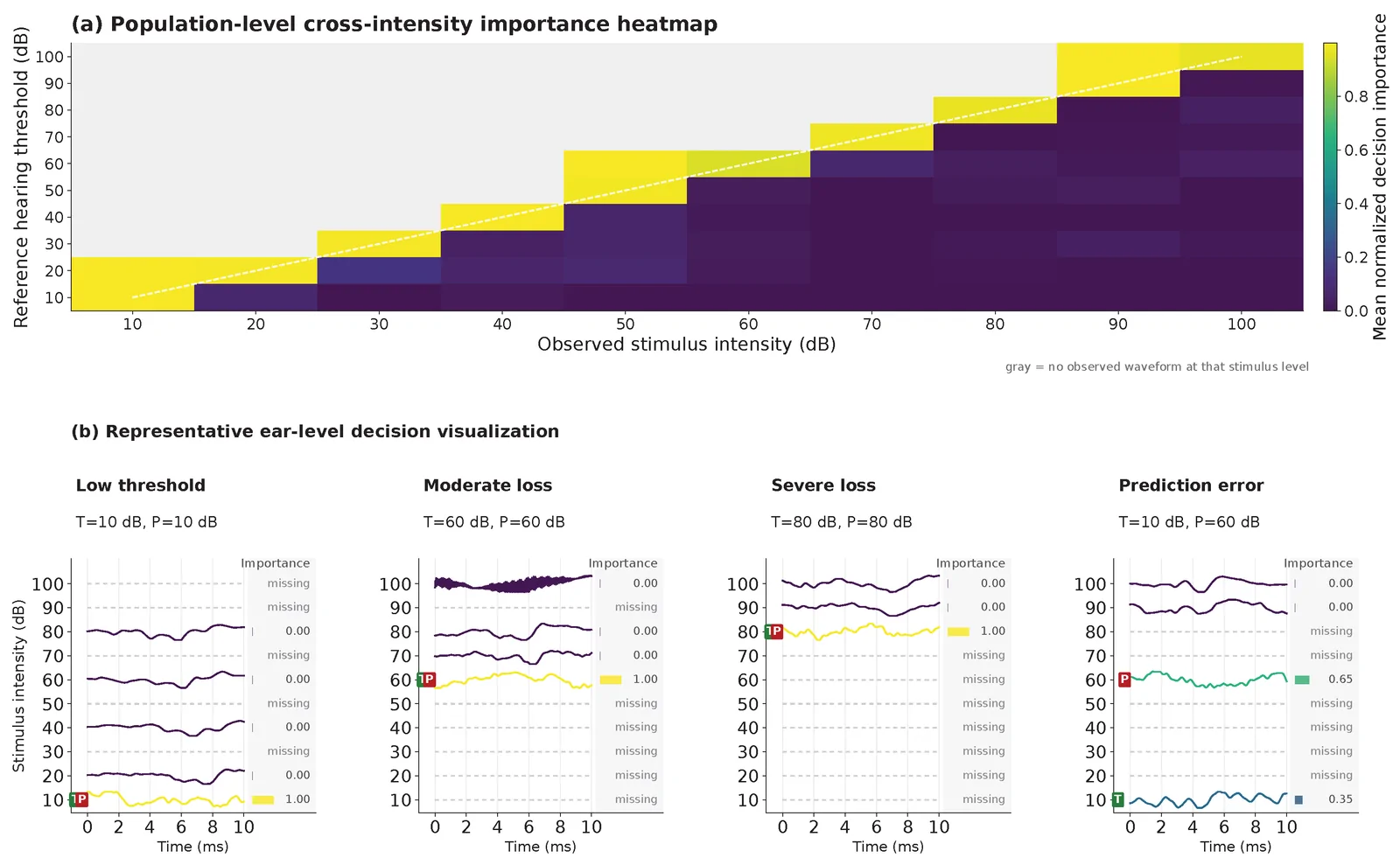
Interpretation of ABR-GraphDoseNet. (**a**) Population importance across observed stimulus intensity and recorded threshold; gray cells indicate that no waveform was observed for that combination. (**b**) Representative ear-level examples spanning correct low, moderate, and severe threshold estimates, together with one prediction error. T and P denote the recorded and predicted thresholds, respectively. Waveform colors encode the normalized decision importance of the corresponding stimulus levels, following the same color scale as in panel (**a**). Importance values are normalized decision scores rather than calibrated probabilities.

**Table 1:** Comparison with Deep Learning Baselines Under Ten-Fold Evaluation Grouped by Subject.

Model	MAE (dB)	RMSE (dB)	Exact (%)	Acc@10 (%)
1D CNN	1.826 ± 0.633	6.728 ± 1.647	89.68 ± 2.71	95.88 ± 2.08
BiLSTM	1.545 ± 0.527	6.345 ± 1.964	90.99 ± 2.04	97.24 ± 1.58
Transformer	1.313 ± 0.308	5.935 ± 1.279	92.39 ± 1.63	97.58 ± 0.78
ABR-GraphDoseNet	**0.896 ± 0.272**	**4.332 ± 1.256**	**93.80 ± 1.41**	**98.64 ± 1.01**

Values are mean ± standard deviation over ten folds grouped by subject. Bold values indicate the best performance. Acc@5 is omitted because steps of 10 dB make it equivalent to exact accuracy.

**Table 2:** Ablation Performance Across Ten Folds Grouped by Subject.

Model Variant	MAE (dB)	RMSE (dB)	Exact (%)	Acc@10 (%)
Full ABR-GraphDoseNet	0.896 ± 0.272	4.332 ± 1.256	93.80 ± 1.41	98.64 ± 1.01
w/o adaptive edges	1.294 ± 0.325	5.563 ± 1.081	92.05 ± 1.93	97.48 ± 0.77
w/o boundary decoder	1.143 ± 0.265	5.201 ± 0.756	92.49 ± 2.02	98.16 ± 0.60
w/o morphology tokenizer	1.570 ± 0.444	6.158 ± 1.058	90.99 ± 2.51	96.17 ± 1.49
GCN instead of GAT	1.467 ± 0.691	6.027 ± 2.184	91.57 ± 2.89	96.85 ± 2.07
w/o global threshold head	2.156 ± 0.999	8.879 ± 3.534	90.50 ± 2.30	95.69 ± 1.60

**Table 3:** Observation-Pattern Analysis Under Subject-Grouped Ten-Fold Evaluation.

Model	MAE (dB)	Acc@10 (%)
Observation-mask-only	1.978	94.95
Full model w/o explicit mask encoding	1.541	96.51
Full model	0.896	98.64

## Data Availability

Restrictions apply to the availability of these data. The dataset used in this study was previously described in Zhang et al., “FIGNet: A Robust and Interpretable Fuzzy-Irreversible Gated Network for Auditory Brainstem Response Classification” [33]. The data were obtained from Shenzhen Children’s Hospital and are available from the corresponding authors upon reasonable request and with the permission of Shenzhen Children’s Hospital, subject to applicable ethical and privacy restrictions.
